# Vacuum-assisted decellularization: an accelerated protocol to generate tissue-engineered human tracheal scaffolds

**DOI:** 10.1016/j.biomaterials.2017.02.001

**Published:** 2017-04

**Authors:** Colin R. Butler, Robert E. Hynds, Claire Crowley, Kate H.C. Gowers, Leanne Partington, Nicholas J. Hamilton, Carla Carvalho, Manuela Platé, Edward R. Samuel, Alan J. Burns, Luca Urbani, Martin A. Birchall, Mark W. Lowdell, Paolo De Coppi, Sam M. Janes

**Affiliations:** a Lungs for Living Research Centre, UCL Respiratory, University College London, London, UK; b Stem Cell and Regenerative Medicine Section, UCL Institute of Child Health and Great Ormond Street Hospital, London, UK; c Department of Haematology, Royal Free Hospital and University College London, London, UK; d Department of Clinical Genetics, Erasmus MC, Rotterdam, Netherlands; e UCL Ear Institute, The Royal National Throat Nose and Ear Hospital, London, UK

**Keywords:** Tissue engineering, Trachea, Transplantation, Tissue scaffolds, Epithelial cells

## Abstract

Patients with large tracheal lesions unsuitable for conventional endoscopic or open operations may require a tracheal replacement but there is no present consensus of how this may be achieved. Tissue engineering using decellularized or synthetic tracheal scaffolds offers a new avenue for airway reconstruction. Decellularized human donor tracheal scaffolds have been applied in compassionate-use clinical cases but naturally derived extracellular matrix (ECM) scaffolds demand lengthy preparation times. Here, we compare a clinically applied detergent-enzymatic method (DEM) with an accelerated vacuum-assisted decellularization (VAD) protocol. We examined the histological appearance, DNA content and extracellular matrix composition of human donor tracheae decellularized using these techniques. Further, we performed scanning electron microscopy (SEM) and biomechanical testing to analyze decellularization performance. To assess the biocompatibility of scaffolds generated using VAD, we seeded scaffolds with primary human airway epithelial cells *in vitro* and performed *in vivo* chick chorioallantoic membrane (CAM) and subcutaneous implantation assays. Both DEM and VAD protocols produced well-decellularized tracheal scaffolds with no adverse mechanical effects and scaffolds retained the capacity for *in vitro* and *in vivo* cellular integration. We conclude that the substantial reduction in time required to produce scaffolds using VAD compared to DEM (approximately 9 days vs. 3–8 weeks) does not compromise the quality of human tracheal scaffold generated. These findings might inform clinical decellularization techniques as VAD offers accelerated scaffold production and reduces the associated costs.

## 1 Introduction

Tracheal tissue engineering has been at the forefront of efforts to generate bioengineered organs because of the lack of therapeutic options available for patients with severe tracheal disease [1], [2], [3], [4], [5]. Both decellularized and synthetic scaffolds have been transplanted in compassionate-use cases [6], [7], [8], [9], [10] and subsequent work has turned to improving the functionality of these transplants. Techniques to improve the quality of cells for transplantation, either through adult stem cell isolation and expansion [11] or induced pluripotent stem (iPS) cell technology [12], [13], [14], [15], [16], are being investigated but the scaffolds onto which cells are seeded also need to be refined. There is currently little consensus on the optimal scaffold for tracheal bioengineering purposes with decellularized [6], [8], synthetic [17] and hybrid stent-based [18] scaffolds at various stages of development.

Clearly, a scaffold that closely mimics normal airway physiology in terms of structure and function is highly desirable [19]. For this reason, decellularization of donor organs is an attractive approach in tracheal bioengineering because the full complexity of human tracheal architecture, extracellular matrix (ECM) and regional structural variation are not yet mimicked by synthetic scaffolds [20]. Ideally, these properties should be retained whilst simultaneously removing all donor cells from the trachea to avoid complications involving immune reaction and rejection after transplantation [21], [22]. Achieving this delicate balance between cell removal and ECM ultrastructure disruption causes decellularization protocols to take longer than may be ideal for the clinical needs of some tracheal stenosis patients. Clinically applied protocols have required many weeks for decellularization [8], [10], [23], a timeframe that rules out the use of a decellularized scaffold in more urgent clinical scenarios such as tracheal agenesis or atresia where the entire trachea is absent and full replacement is required soon after birth [24], [25].

**Fig. 1:**
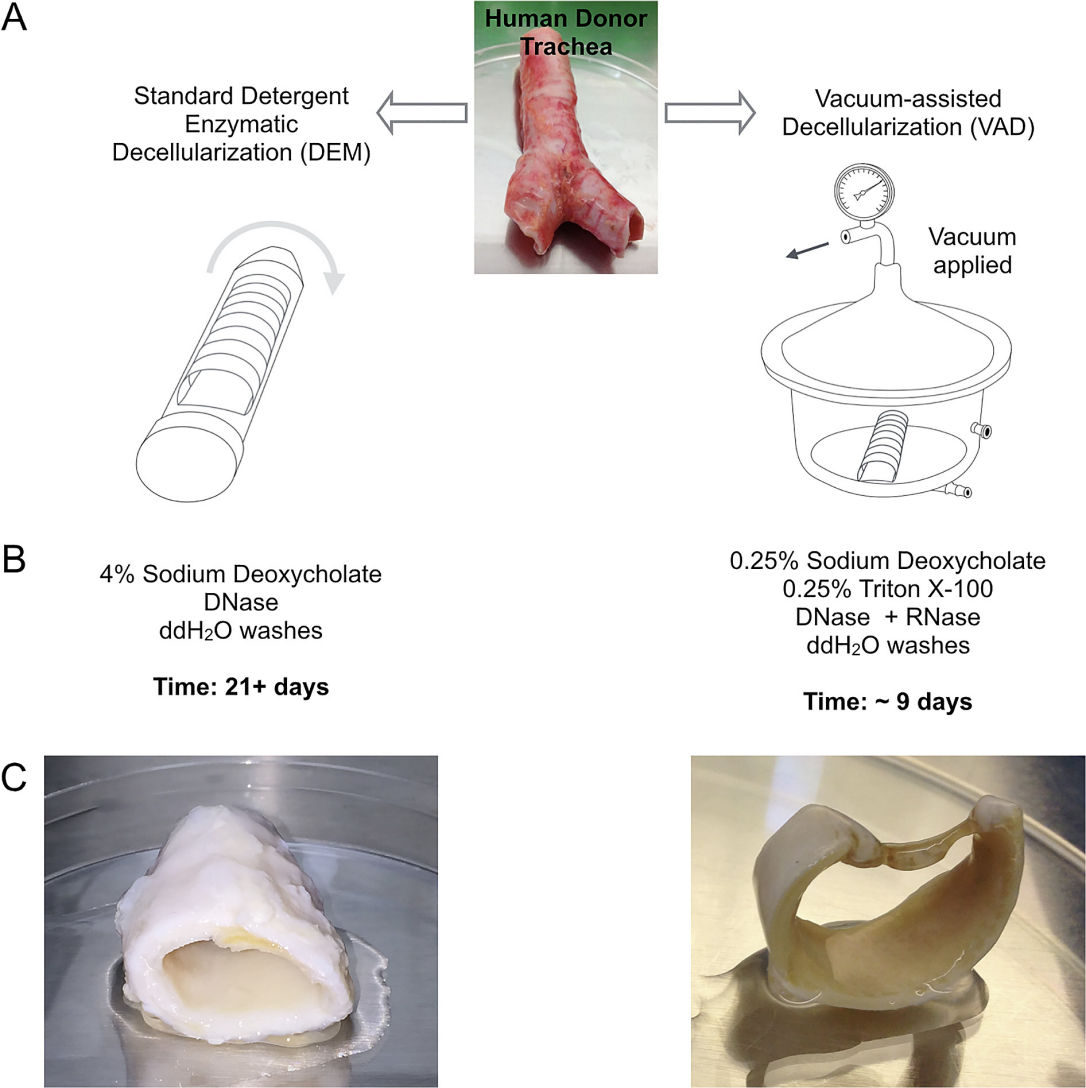
**Schematic comparison of the human tracheal decellularization techniques compared in this study**. (A) This study compares the clinically applied detergent-enzymatic method (DEM; left) with a vacuum-assisted decellularization (VAD; right) protocol designed to reduce the time taken to produce scaffolds and to reduce the costs associated with clinical-grade scaffold production. (B) Outline of protocols applied to tracheal segments. (C) Macroscopic appearance of tracheal scaffolds following decellularization.

Methods to accelerate the decellularization process are being developed [26] but as yet no direct comparison of the scaffolds produced by different methods has been performed using human tracheae, particularly in terms of their *in vivo* responses [27]. We developed an accelerated vacuum-assisted decellularization (VAD) protocol, which reduces the time required for decellularization from several weeks [8], [10], [28] to just nine days (Fig. 1). We hypothesized that VAD would effectively decellularize human tracheal scaffolds and that the resulting scaffolds would retain similar biomechanical properties and key extracellular matrix components to those obtained with the clinically applied detergent-enzymatic method (DEM). Further, we hypothesized that both VAD and DEM scaffolds would support the engraftment of primary human airway epithelial cells *in vitro* and would vascularize to similar extents following *in vivo* implantation.

## 2 Materials and methods

### 2.1 Decellularization of human tracheae

#### 2.1.1 Tissue procurement

Human tracheae were obtained from cadaveric donors (30–80 years age) identified through the National Health Service Blood and Transplant (NHSBT) tissue retrieval team, by agreement and with appropriate consent, following the current clinical practice for non-living tissue retrieval in the U.K. Ethical approval was obtained through the National Research Ethics Committee (REC reference 11/LO/1522). Tracheae were retrieved less than 48 h post mortem and were removed in their entirety from cricoid to carina. Donor tracheae were immediately rinsed in 1 L 0.9% normal saline and surrounding tissue was dissected away. The tracheae were immersed in 20% chlorhexidine solution for 5 min followed by a further three rinses in 0.9% saline. Intact tracheae were divided into two equal halves and these matched donor pairs were processed for either the clinically applied standard protocol [23] of repeated cycles of detergent and enzyme (detergent-enzymatic method; DEM) or frozen and then decellularized using the vacuum-assisted decellularization (VAD) protocol [26] such that decellularization using both procotols finished simultaneously. A matched donor design was chosen due to the difficulties in obtaining large numbers of human donor organs for research purposes and to minimize the effect of inter-individual variability on results.

#### 2.1.2 Detergent-enzymatic method (DEM) decellularization

Tracheae were subjected to 25 cycles of distilled water for 72 h at 4 °C, 4% sodium deoxycholate (Sigma) for 4 h and 2000 kU DNase I in 1 M sodium chloride (Sigma) for 3 h. Tracheae were continuously rotated on a roller throughout the decellularization process and were stored at 4 °C in PBS until use.

#### 2.1.3 Vacuum-assisted decellularization (VAD)

Tracheae to be processed by VAD were aspirated dry and frozen at −80 °C. Tissue was thawed to room temperature for 24 h and rinsed in PBS. Decellularization was performed in an autoclaved stainless steel Ricordi chamber. Luer locks with 3-way ports and a pressure gauge were attached to the chamber such that a vacuum (1 Torr) could be generated. All decellularization steps were performed under vacuum on a tissue agitator at 140 rpm at 37 °C unless otherwise indicated. All solutions were supplemented with 1% penicillin/streptomycin (Sigma). Tracheae were incubated in detergent solution containing 0.25% sodium deoxycholate (Sigma) and 0.25% Triton X-100 (Sigma) in HBBS for 24 h at 37 °C. Tracheae were then rinsed twice in sterile water for 2 h and incubated for a further 44 h in sterile water at 4 °C on a tissue agitator at 20 rpm. Following the wash step, tracheae were incubated in DNase I (2 kU/ml) and RNase (4 U/ml) at 37 °C for 24 h, followed by another 24 h wash step in sterile water at 4 °C as described above. The DNase/RNase step and wash was repeated once prior to a final wash step of sterile water for 48 h at 4 °C as described above. Tracheae were then stored at 4 °C in PBS until use.

### 2.2 Scaffold characterization

#### 2.2.1 Immunohistochemistry and immunofluorescence

Samples were fixed in 4% paraformaldehyde (PFA) overnight prior to standard processing for paraffin embedding. Serial paraffin sections were cut at 5 μm thickness. Haematoxylin and eosin, Periodic acid-Schiff, picrosirius red and Masson’s trichrome staining were performed on sections using an automated staining system (Tissue-Tek). For immunofluorescence staining, slides were dewaxed using an automated protocol and antigen retrieval was performed using citrate buffer (pH 6.0). Primary antibodies against cytokeratin 5 (CK5; Abcam; ab17130), collagen IV (COL-94; Abcam; ab6311) and laminin (Sigma; L9393) were used. Species-appropriate secondary antibodies conjugated to AlexaFluor dyes (Molecular Probes) were then used. Images were acquired using a Zeiss LSM700 confocal microscope. For immunohistochemistry, slides were dewaxed and antigen retrieved as for immunofluorescence. Primary antibodies against CD45 (Abcam; ab10558), endomucin (V.7C7.1; Abcam; ab106100) and F4/80 (Serotec; MCA497GA) were used. Sections were then incubated with species-appropriate biotinylated F(ab’)2 for 30 min, developed using ABC reagent and superDAB (Dako) and finally counterstained with haematoxylin.

#### 2.2.2 Whole-mount immunocytochemistry

For whole-mount imaging, cell-seeded scaffolds were fixed in 4% PFA for 30 min and blocked for 1 h in blocking buffer (10% fetal bovine serum in PBS containing 0.01% Triton X-100). CK5 primary antibody (Abcam; ab17130) incubation was overnight at 4 °C in blocking buffer without Triton X-100. Scaffolds were washed three times in PBS for 5 min and species-appropriate secondary antibody (AlexaFluor dyes; Molecular Probes) was added for 2 h at room temperature. Scaffolds were washed three times in PBS and DAPI (1:10,000 in PBS) was applied to counterstain scaffolds before imaging. Scaffolds were oriented luminal-side down on a low-profile chamber-well slide (Ibidi; #80826) for image acquisition using a LSM700 confocal microscope (Zeiss).

#### 2.2.3 DNA quantification

Isolation of DNA from tracheal samples was performed using a PureLink DNA extraction kit (Invitrogen) according to the manufacturer’s instructions. Briefly, samples were digested overnight in proteinase K and DNA was isolated using the supplied buffers and mini-columns. Quantification of eluted DNA was carried out by spectrophotometry using a NanoDrop 8000 (Thermo Scientific). All samples had a 260/280 ratio of between 1.8 and 2.0.

#### 2.2.4 Extracellular matrix quantification

Collagen, elastin and glycosoaminglycans were quantified as previously described [29]. Collagen content was determined using a total collagen assay kit (QuickZyme Biosciences) according to the manufacturer’s instructions. Representative whole tracheal samples of native and decellularized trachea were weighed and directly hydrolyzed in 6 M HCl for 20 h at 95 °C. Samples were allowed to cool to room temperature and centrifuged to remove debris. To detect hydroxyproline residues, supernatants were mixed with a chromogen solution for 1 h at 60 °C. Absorbance for each sample was read using a colorimetric reader (555 nm). Standards were generated by serial dilution of rat tail collagen I (BD Biosciences) from 300 μg/ml.

Elastin quantification was performed using an elastin assay kit (FASTIN, Biocolor). Representative whole tracheal samples of both native and decellularized tissue were homogenized and solubilized twice with 0.25 M oxalic acid incubated at 95 °C. Extracts were combined with FASTIN dye reagent (5,10,15,20-tetraphenyl-21H,23H-porphine tetrasulfonate (TPPS)) and the absorbance was determined at 555 nm. Concentrations were determined using α-laminin standards.

The sulphated glycosaminoglycan (sGAG) content was quantified according to the Blyscan GAG Assay Kit (Biocolor). Representative wet tissue samples were incubated in 1 ml of papain digestion buffer at 65 °C for 18 h. Aliquots of each sample were mixed with 1,9-dimethyl-methylene blue dye and the reagents from the GAG assay kit. The absorbance was read at 656 nm and quantified according to standards generated from bovine tracheal chondroitin-4-sulphate.

#### 2.2.5 Scanning electron microscopy

Scanning electron microscopy (SEM) images show the luminal surface of decellularized human donor tracheal scaffolds. Scaffolds were fixed in 2.5% glutaraldehyde in 0.1 M phosphate buffer, washed with 0.1 M phosphate buffer (pH 7.4), post fixed with 1% osmium tetraoxide and 1.5% potassium ferrocyanine in 0.1 M phosphate buffer and rinsed with distilled water. Specimens were then dehydrated to 100% ethanol using a graded ethanol-water series and critical-point dried using carbon dioxide. Samples were mounted onto aluminum stubs with sticky carbon tabs. A Gatan ion beam coater was used to coat scaffolds with a layer of Au/Pd (approx. 2 nm thickness). Images were obtained using a 7401 FEG scanning electron microscope (Jeol, USA).

#### 2.2.6 Mechanical testing

All decellularized tracheal samples were equilibrated to room temperature prior to testing and kept hydrated in PBS for mechanical testing. Each trachea was cut into 3 cm sections and compressed with antero-posterior and lateral compressive forces. The load (N) exerted by the scaffold was calculated when the scaffold was compressed to 50% of the starting diameter. Further tracheal samples were cut into dumbbell sections with a template cutter such that vertical (axial) forces across tracheal rings and trachealis could be applied. A further dumbbell section was horizontally cut across a tracheal ring. The load required to pull apart the sample until breaking point was measured using an Instron 5565 tensile tester (Instron Ltd., UK). The tensile stress versus strain properties were assessed at a displacement rate of 50–100 mm/min. The Young’s modulus is a measure of material stiffness and was calculated using Bluehill software (Instron, U.K.) as the slope of the straight line portion between 0 and 5 mm.

### 2.3 Epithelial cell seeding and tracking on decellularized scaffolds

#### 2.3.1 Primary epithelial cell seeding

Primary human bronchial epithelial cells (HBECs) were obtained either by enzymatic digestion from donor human trachea or from explant biopsy outgrowth [30]. Primary HBECs were maintained for up to three passages in bronchial epithelial growth medium (BEGM; Lonza, UK) as previously described [31]. Decellularized human tracheal scaffolds were cut into 5 mm discs using a biopsy punch, such that they fitted into 96-well plate wells. Prior to epithelial seeding, scaffolds were oriented with luminal/mucosal-surface side up in a low-adhesion 96-well plate (Nunc) and incubated overnight in 200 μl of BEGM medium. Epithelial cells were seeded at 0.25 × 10^6^ cells/cm^2^, 0.5 × 10^6^ cells/cm^2^ or 1.0 × 10^6^ cells/cm^2^ and allowed to adhere overnight. Scaffolds were transferred to new low-adhesion plates with the same orientation maintained. Subsequently, medium was changed every 48 h.

#### 2.3.2 Generation of luciferase-tagged immortalized HBECs

Immortalized human bronchial epithelial cells (iHBECs; a gift from Dr. Gisli Jenkins, University of Nottingham, U.K.) were transduced with mStrawberry-Luc lentivirus to create a stable iHBEC cell line co-expressing mStrawberry fluorescent protein and luciferase. Lentivirus production was as previously described [32] but created with the pLV-LS1 transfer vector and the packaging and envelope vectors pCMV-dR8.74 and pMD.G2 (Plasmid Factory). Briefly, viral supernatants were created by co-transfecting 293T HEK cells with the above plasmids using JetPEI (Polyplus Transfection). Supernatant containing lentivirus was collected at 48 and 72 h and was concentrated by ultracentrifugation. Each collection underwent ultracentrifugation to concentrate virus. To generate a transduced cell line, iHBECs were grown in LHC9 medium (Gibco), seeded at 1 × 10^5^ cells in a T25 flask and transduced at an MOI of 7.5 with 4 μg/ml polybrene (Sigma). After 8 h, medium was changed and cells were passaged when approximately 80% confluent. To obtain a pure luciferase-expressing population, mStrawberry fluorescent protein-expressing cells were sorted by fluorescence-activated cell sorting (FACS) using a BD Aria flow cytometer.

#### 2.3.3 In vitro cell tracking using IVIS imaging

IVIS imaging (Caliper Life Sciences) was used as a non-invasive *in vitro* method of tracking luciferase-tagged iHBECs on decellularized human tracheal scaffolds by detecting a luminescence signal from cells in the presence of the luciferase substrate luciferin. Scaffolds were fashioned into 5 mm discs and placed into 96-well plate wells with the luminal surface facing upwards. Scaffolds were incubated in LHC9 medium (Gibco) overnight at 37 °C and 5% CO_2_. Cells were seeded on the scaffolds and transferred to a new low adhesion plate prior to imaging. D-luciferin was added to the medium at 150 μ-g/ml and scaffolds were imaged within the plates at position ‘C’ using automatic acquisition times.

### 2.4 *In vivo* scaffold integration

#### 2.4.1 Chick chorioallantoic membrane (CAM) assay

Fertilized chicken eggs (Henry Stewart and Co., UK) were incubated at 37 °C. After 3 days of incubation, an approximately 3 cm diameter window was cut into the shell using dissecting scissors, revealing the embryo and CAM vasculature. Windows were sealed with tape and eggs were returned to the incubator for 5 days. At day 8 of incubation, decellularized human tracheal scaffolds (approximately 2–3 mm^3^) and pieces of absorbable gelatin sponge (Gelfoam; Pfizer; a negative control) were placed on the CAM between blood vessels. Scaffolds were checked daily for 6 days, when scaffolds were photographed *in ovo* with a stereomicroscope (Leica). Vessel ingrowth was quantified blindly as previously described [33].

#### 2.4.2 Xenotransplantation of decellularized human trachea

Human tracheal scaffolds were prepared as described above and cut into full thickness 5–7 mm^2^ pieces, irradiated with 10,000 Gy to ensure sterility and stored in PBS prior to implantation. Adult CD1 mice were used for subcutaneous implantation of scaffolds. Live animal work was ethically approved and carried out under Home Office Project Licence PPL 70/7504. Briefly, CD1 mice were anaesthetized with a 2–5% isoflorane:oxygen gas mix for induction and maintenance. Under aseptic conditions, the dorsum of the mouse was shaved and chlorhexidine was applied to the skin. 5 mm transverse incisions over each dorsal flank were made and subcutaneous pockets created. One scaffold was inserted in each pocket and these were closed with buried 4/0 Vicryl interrupted sutures. Scaffolds were implanted for 56 days and the experiments were terminated accordingly. Scaffolds were retrieved *en bloc* for histopathological analysis.

### 2.5 Statistical analysis

Results are presented as mean ± standard deviation. Data were analyzed in Microsoft Excel and GraphPad Prism 6.0. Statistical tests are indicated in figure legends and significance was accepted at the 5% level.

## 3 Results

### 3.1 VAD decellularizes human tracheae with similar efficacy to the clinically applied DEM

#### 3.1.1 Histological assessment

**Fig. 2:**
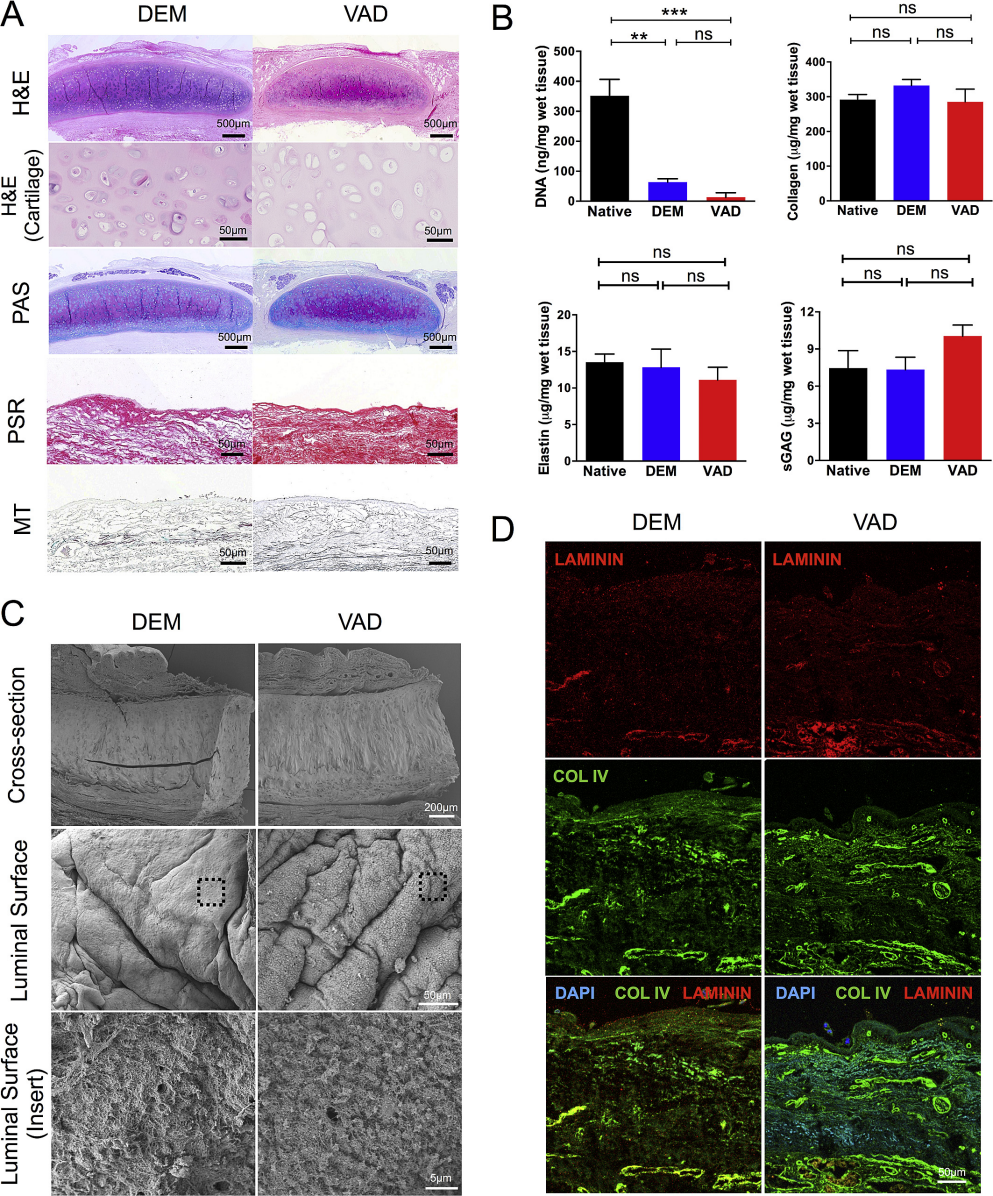
**Human donor trachea is efficiently decellularized by the accelerated vacuum-assisted decellularization (VAD) protocol**. (A) The histological appearance of human tracheae decellularized using both the clinically applied detergent-enzymatic method (DEM; left) and a vacuum-assisted decellularization protocol (VAD; right) are shown using haematoxylin and eosin (H&E), periodic acid-Schiff [15], picrosirius red (PSR) and Masson’s trichrome (MT) staining. Scale bars = 500 μm or 50 μm, as indicated. (B) Decellularized tissue composition was analyzed by comparing DNA, collagen, elastin and sulphated glycosaminoglycan (sGAG) content with native human trachea. Six donor matched tracheae were assessed and data were analyzed using a Kruskal-Wallis test (ns = non-significant, ** = p < 0.01, *** = p < 0.001). (C) Scanning electron microscopy (SEM) images of human tracheae decellularized using DEM or VAD. Upper panel scale bar = 200 μm; middle panel = 50 μm; lower panel = 5 μm. (D) Immunofluorescence staining of DEM- or VAD-prepared human trachea using antibodies against laminin (red) and collagen IV [12] and counterstained with DAPI (blue). Scale bar = 50 μm. (For interpretation of the references to colour in this figure legend, the reader is referred to the web version of this article.)

To assess the efficacy of vacuum-assisted decellularization (VAD) we compared this protocol with the clinically applied detergent-enzymatic method (DEM). Histologically, VAD removed nuclei from the epithelium, the submucosal glands and the tracheal cartilage but cartilage lacunae remained intact in both scaffolds (Fig. 2A). Periodic acid-Schiff (PAS) staining demonstrated the widespread retention of proteoglycans and glycoproteins in cartilage and also in submucosal glands, which may reflect the protection of the glandular basement membrane during decellularization (Fig. 2A). Picrosirius red (PSR) staining demonstrated the widespread retention of collagen I and III fibers within tracheal scaffolds decellularized using both DEM and VAD, findings supported by Masson’s trichrome (MT) staining (Fig. 2A).

#### 3.1.2 Scaffold composition

Reflecting the extensive decellularization by both protocols, the DNA content of scaffolds was significantly reduced in comparison to native trachea (Fig. 2B). While there was a trend towards lower DNA content in VAD tracheae as compared to DEM tracheae, consistent with results following the application of vacuum pressure to porcine tracheal decellularization [26], this did not reach statistical significance. However, the abundance of structural components of scaffolds such as elastin, collagen and sulphated glycosaminoglycans (sGAGs) was not significantly affected by either DEM or VAD decellularization (Fig. 2B).

#### 3.1.3 Scanning electron microscopy

Scanning electron microscopy (SEM) of decellularized scaffolds confirmed the absence of cells in both DEM and VAD scaffolds and showed the folded macroarchitecture characteristic of human trachea. At higher magnification, a meshwork of fibers was visible on the luminal surface of both scaffolds (Fig. 2C). However, the basement membrane proteins laminin and collagen IV were not found at the luminal surface using immunofluorescence but were retained deeper within scaffolds (Fig. 2D).

### 3.2 Biomechanical properties of human tracheae are retained following decellularization

**Fig. 3:**
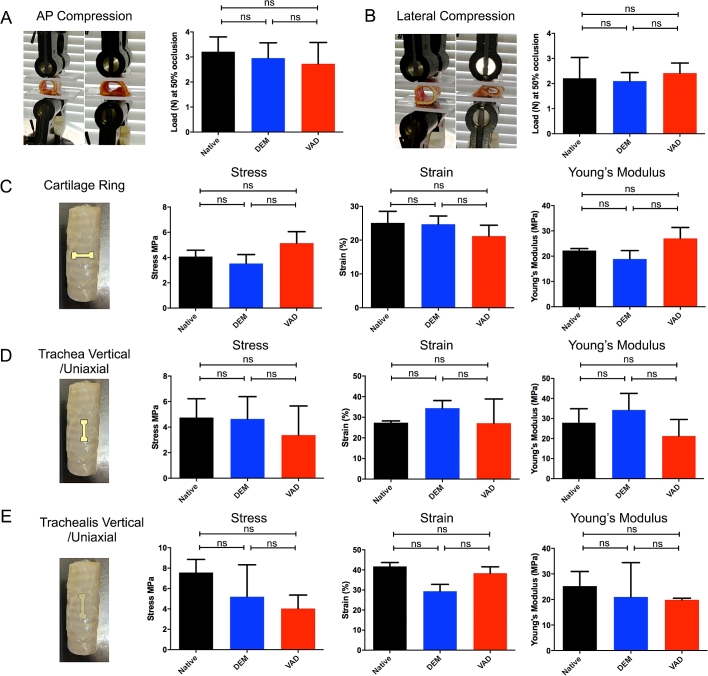
**The biomechanical properties of human donor tracheae are retained following decellularization using an accelerated vacuum-assisted decellularization (VAD) protocol**. (A) Anterior-posterior (AP) compression. (B) Lateral compression. (C) Cartilage ring stress, strain and Young’s modulus. (D) Trachea (vertical/uniaxial) ring stress, strain and Young’s modulus. (E) Trachealis (vertical/uniaxial) ring stress, strain and Young’s modulus. 4 donor tracheae in at least technical triplicates were analyzed using a Kruskal-Wallis test (ns = non-significant).

An important aspect of tracheal bioengineering is the structural integrity of scaffolds post-transplantation. To assess whether VAD compromises scaffold mechanical properties, we compared these scaffolds with DEM-decellularized and native tracheae. We found that decellularization did not significantly affect a scaffold’s ability to resist compression in either the anterior-posterior (Fig. 3A) or lateral (Fig. 3B) axes. Further, the elastic properties of the cartilage rings (Fig. 3C), the tracheal wall (Fig. 3D) and the trachealis (Fig. 3E) were not significantly affected by VAD or DEM decellularization.

### 3.3 Cytocompatibility of decellularized human trachea following decellularization

#### 3.3.1 Epithelial cell attachment to decellularized scaffolds

**Fig. 4:**
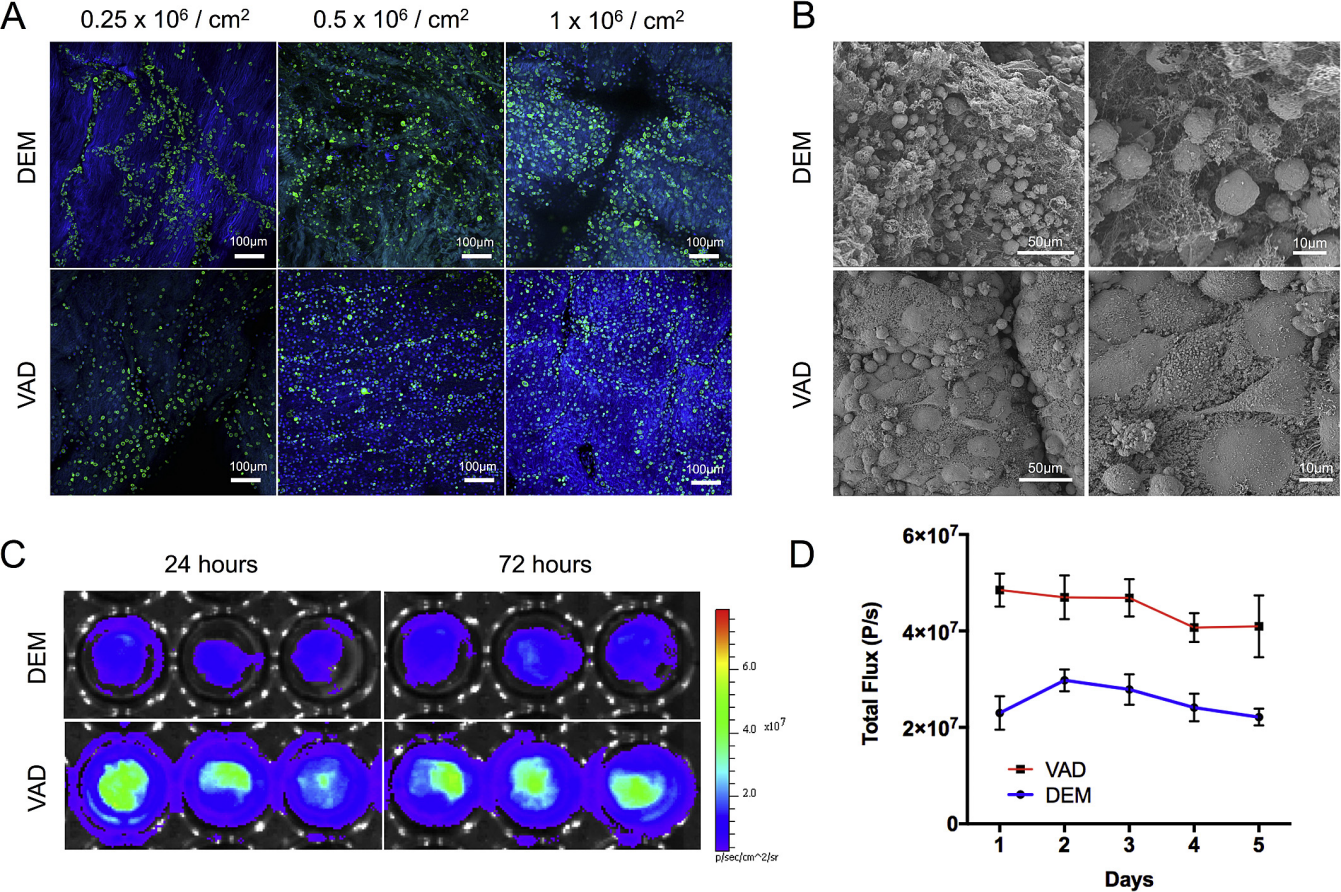
**Primary human airway epithelial cell seeding of decellularized human donor trachea demonstrates cytocompatibility**. (A) Primary human bronchial epithelial cells (HBECs) seeded onto either detergent-enzymatic (DEM) or vacuum-assisted decellularization (VAD)-decellularized human tracheal scaffolds. Scaffolds were fixed at 72 h, stained using an antibody against the airway basal cell marker cytokeratin 5 (CK5; green) and counterstained with DAPI (blue). Scale bars = 100 μm. (B) Scanning electron microscopy images show the morphology of primary HBECs on scaffolds after 72 h. Scale bar = 50 μm (left) and 10 μm (right). (C) Bioluminescence imaging of luciferase-tagged immortalized human bronchial epithelial cells (iHBECs) demonstrates the comparable survival of cells after 72 h on decellularized scaffolds *in vitro*. (D) Quantification of bioluminescence imaging of iHBECs seeded at 1 × 10^6^ cells/cm^2^ (n = 3 technical triplicates; representative of three independent experiments) over 5 days for both DEM- and VAD-decellularized scaffolds. (For interpretation of the references to colour in this figure legend, the reader is referred to the web version of this article.)

For VAD-decellularized tracheae to be of use clinically, the scaffolds must retain the capacity to support cells in addition to retaining their mechanical properties. As airway epithelial cells represent a key functional aspect of bioengineered tracheae [34], [35], we examined the adhesion of primary human bronchial epithelial cells (HBECs) to the luminal surface of DEM- and VAD-prepared scaffolds *in vitro*. By immunofluorescence staining 72 h post-seeding, we observed that HBECs adhered well to both scaffolds but that high seeding densities improved the retention of cells in both types of scaffold (Fig. 4A). Scanning electron microscopy (SEM) of scaffolds seeded at 1 × 10^6^ cells per cm^2^ also demonstrated that cells had adhered to both grafts and revealed possible morphological differences between DEM- and VAD-prepared scaffolds with cells more rounded on DEM scaffolds and flatter on VAD scaffolds, a possible indication of more mature cellular adhesions to scaffolds in the VAD condition (Fig. 4B).

#### 3.3.2 Cell survival on decellularized scaffolds using luciferase-tagged epithelial cells

In order to monitor cells in real time on decellularized scaffolds, we monitored the bioluminescence of luciferase-transduced immortalized human bronchial epithelial cells (iHBECs) using an *in vivo* imaging system (IVIS). Non-invasive monitoring of cells on the decellularized scaffolds using this system demonstrated a stable signal over the 5-day period. VAD scaffolds had higher signals throughout this experiment, apparently caused by higher initial engraftment of cells on VAD scaffolds compared to DEM scaffolds (Fig. 4C and D). However, we cannot rule out that differences between the decellularization protocols, such as enzyme concentration, affected these results as scaffolds were stored identically after decellularization and optimization of this step may be necessary for optimal cell seeding.

### 3.4 *In vivo* behavior of DEM/VAD-decellularized tracheal scaffolds

#### 3.4.1 Chorioallantoic membrane assays

**Fig. 5:**
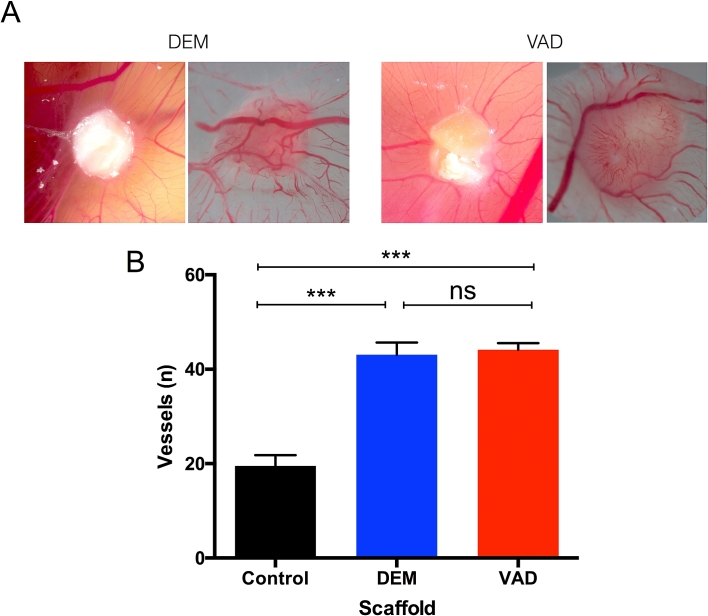
**Acellular human tracheal scaffolds are pro-angiogenic *in vivo***. (A) To assess the capacity of scaffolds for vascularization, detergent-enzymatic (DEM) or vacuum-assisted decellularized (VAD) human donor tracheal scaffolds were transplanted into a chick chorioallantoic membrane (CAM) assay. Examples of DEM- and VAD-prepared scaffolds 8 days post implantation are shown. (B) Macroscopic quantification of vessels converging on the scaffolds was performed for at least six replicates of each scaffold type. Polyester membrane served as a negative control. Data were analyzed using a Kruskal-Wallis test (ns = non-significant, *** = p < 0.001; for decellularized scaffolds, three donors were analyzed in technical triplicate; for controls, n = 6).

For cells seeded *ex vivo* to survive following transplantation, there is a clear need for rapid integration and angiogenesis in implanted decellularized scaffolds. To assess the potential of DEM- and VAD-prepared scaffolds for vascular integration, we performed chick chorioallantoic membrane (CAM) assays [29], [36] using fragments of either DEM- or VAD-decellularized human tracheae. Gelfoam^®^ acted as a negative control. Macroscopically, we observed a spoke-wheel pattern of vessels four days after implantation, which suggested that vessels were attracted to both DEM- and VAD-prepared scaffolds (Fig. 5A). Quantification of the number of vessels converging on the decellularized scaffolds revealed that decellularized scaffolds stimulated significantly more vessel ingrowth than negative controls (Fig. 5B) and that the accelerated VAD protocol did not negatively influence vessel ingrowth (Fig. 5A).

#### 3.4.2 Xenotransplantation assays

**Fig. 6:**
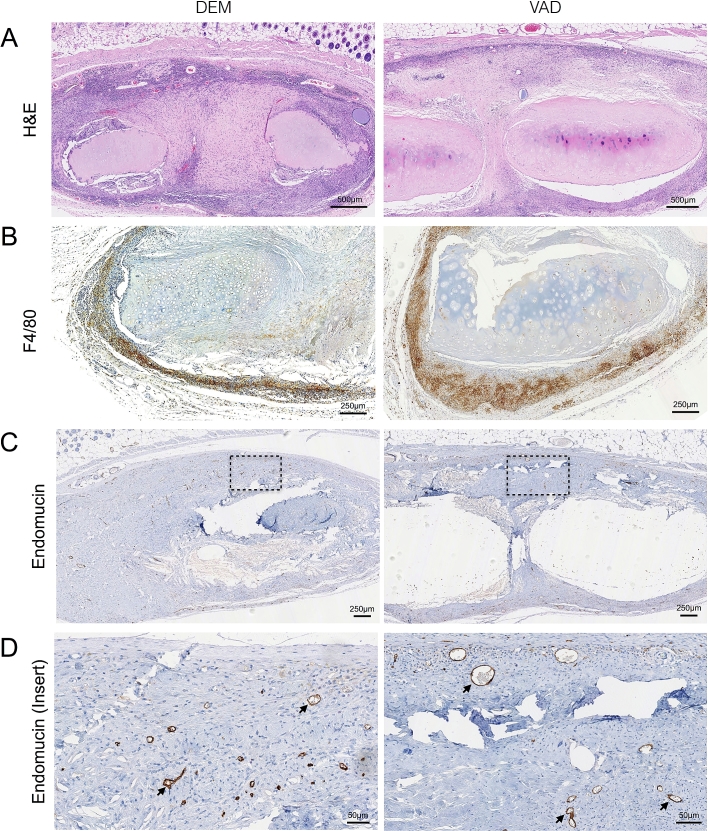
**Biocompatibility and cellular integration of decellularized human tracheal scaffolds in a subcutaneous transplantation model**. Detergent-enzymatic (DEM) or vacuum-assisted decellularized (VAD) human donor tracheal scaffolds were implanted subcutaneously (n = 4) in CD1 mice and evaluated after 8 weeks. (A) Haematoxylin and eosin (H&E) staining of representative recovered scaffolds are shown. Scale bars = 500 μm. Immunohistochemical staining was carried out using antibodies against (B) the macrophage marker F4-80 (scale bars = 250 μm) and (C) the endothelial cell marker endomucin to show neovascularization within the implanted scaffolds (scale bars = 250 μm). (D) Higher magnification of the insert shown in (C). Scale bars = 50 μm.

Finally, we compared the behavior of DEM- and VAD-prepared human tracheal scaffolds in a xenotransplantation assay. 3 mm^3^ pieces of either DEM- or VAD-prepared tracheae were implanted subcutaneously into immunocompetent CD1 mice and retrieved after 8 weeks. Throughout the experiment the mice behaved normally without evidence of inflammation, extrusion of the implanted tissue or death. At the time of retrieval, tissue surrounding the implants appeared healthy, without signs of adverse reaction. Histological analyses revealed that DEM- and VAD-prepared tracheal scaffolds showed similar cellular infiltration around the graft (Fig. 6A) and that this was predominantly macrophages (Fig. 6B). Evidence of vascularization was also seen in both scaffold types as demonstrated by endomucin-stained vessels within the grafts (Fig. 6C and D).

## 4 Discussion

We show that a vacuum-assisted decellularization (VAD) protocol produces human tracheal scaffolds with comparable decellularization efficiency, extracellular matrix retention, mechanical properties, cytocompatibility and *in vivo* integration capacity to scaffolds decellularized using the clinically applied detergent-enzymatic method (DEM). However, VAD is an accelerated protocol requiring only 9 days from donor tissue procurement to final decellularized scaffold; by contrast, the DEM method takes 3–8 weeks [8], [10], [23]. The efficacy of decellularization reported here is in line with previously published accepted standards for tissue decellularization [37]. These results are an important step towards the use of decellularized human cadaveric donor tracheae as scaffolds for airway bioengineering because expedited preparation broadens the clinical scenarios in which decellularization is appropriate. The incorporation of a freezing step prior to decellularization is of particular importance as it suggests banking of donor tracheae as a clinically relevant strategy. An important consideration is that current protocols for storage of pre-decellularized tracheae mean their salient properties might deteriorate over time in storage [38] and the optimization of storage conditions for decellularized materials is a priority for the field.

Production of decellularized scaffolds that share similar biomechanical properties to native tissue is an important aim as the tracheal cartilage maintains a patent airway in healthy individuals. Previous studies suggest that decellularization using some protocols could negatively affect the structural properties of porcine tracheae [39]. Here, we comprehensively tested the biomechanical properties of VAD-decellularized human tracheae including lateral and antero-posterior compression finding that the resistance to compression and elastic properties remain similar to that of native human tracheae. It is noteworthy that donor tracheae have quite large variance between samples and this may contribute to non-significant differences found despite controlling for this with matched samples. In the future, particularly for clinical application, donor selection criteria may have to be more stringent than they have been for research purposes.

Scaffold preparation represents the first step in the process of generating a transplantable tissue-engineered airway. Clinically applied protocols have recellularized scaffolds with autologous cells, including epithelial cells, mesenchymal stromal cells (MSCs) and MSC-derived chondrocytes [6], [8], [9], [10]. As such, it is important that, as well as maintaining the expected mechanical properties, scaffolds are able to support the adherence and survival of relevant cell types. Here, we demonstrated that human bronchial epithelial cells (HBECs) attach to and survive on both decellularized human scaffold types *in vitro*. However, neither the DEM or VAD decellularization method preserved the basement membrane that is normally present beneath airway epithelial cells; instead, a mesh of fibers ordinarily constrained to the stromal compartment was presented at the luminal surface [40]. This may explain the high seeding densities of HBECs required for cell retention as many cells may undergo apoptosis before basement membrane production and/or remodeling occurs. We believe that future studies should investigate the importance of the basement membrane in airway bioengineering: basement membrane retention or reconstruction could improve the cytocompatibility of both decellularized and synthetic scaffolds, which presently require huge numbers of HBECs to re-epithelialize [11]. Authors treating severe skin burns with cultured epidermal stem cells found that the application of these cells on a fibrin sheet greatly improved the ‘take rate’ and ease of application [41]. Similar to other tissue- engineered epithelia, it may also be possible to transplant airway epithelium as a pre-prepared sheet product [42], [43] or as organoids [44]. Optimization of protocols for functional airway differentiation, to generate a ciliated epithelium on VAD human scaffolds, as has been demonstrated in rodent tracheal scaffolds [45], is also desirable. Pre-seeding and integration of stromal cells into VAD scaffolds could also be considered [46].

The mechanism, time-course and quality of vascularization of tissue-engineered constructs is presently unknown and requires further research, as highlighted at a recent international consensus meeting [47]. We believe that our data demonstrating comparable behavior of DEM and VAD scaffolds in terms of their ability to promote a rapid angiogenic response in CAM experiments and comparable vascular integration as subcutaneous implants provide reassurance that reducing the time taken to prepare scaffolds using a vacuum system does not have detrimental effects on scaffold performance.

In the trachea, translation of these protocols into clinical practice will require the development of equipment capable of delivering vacuum pressure to GMP standards. While the reagents used in the protocol reported here have previously been adapted towards GMP manufacture, the development of standard operating procedures and quality control measures for VAD will be an important step towards this. Further, the concept of VAD might be useful in clinical protocols to produce decellularized scaffolds from other organs but protocols will likely require customization to each setting.

## 5 Conclusion

This comparison between clinically used DEM decellularization and accelerated vacuum-assisted decellularization (VAD) of human tracheae shows that VAD can rapidly generate decellularized tracheal scaffolds that are comparable to those previously used for clinical transplantation in a number of key aspects. Importantly, this innovative procedure allows for banking of tracheae prior to decellularization for future applications. The similar properties of DEM and VAD scaffolds both *ex vivo* and *in vivo* provide a rationale for the use of VAD in clinical applications of tissue-engineered airway scaffolds.

## References

[1] Atala A., Kasper F.K., Mikos A.G. (2012). Engineering complex tissues. Sci. Transl. Med..

[2] Fishman J.M., Wiles K., Lowdell M.W., De Coppi P., Elliott M.J., Atala A., Birchall M.A. (2014). Airway tissue engineering: an update. Expert Opin. Biol. Ther..

[3] Crowley C., Birchall M., Seifalian A.M. (2015). Trachea transplantation: from laboratory to patient. J. Tissue Eng. Regen. Med..

[4] Haykal S., Salna M., Waddell T.K., Hofer S.O. (2014). Advances in tracheal reconstruction. Plast. Reconstr. Surg. Glob. Open.

[5] Weiss D.J. (2014). Concise review: current status of stem cells and regenerative medicine in lung biology and diseases. Stem Cells.

[6] Elliott M.J., De Coppi P., Speggiorin S., Roebuck D., Butler C.R., Samuel E., Crowley C., McLaren C., Fierens A., Vondrys D., Cochrane L., Jephson C., Janes S., Beaumont N.J., Cogan T., Bader A., Seifalian A.M., Hsuan J.J., Lowdell M.W., Birchall M.A. (2012). Stem-cell-based, tissue engineered tracheal replacement in a child: a 2-year follow-up study. Lancet.

[7] Hamilton N.J., Kanani M., Roebuck D.J., Hewitt R.J., Cetto R., Culme-Seymour E.J., Toll E., Bates A.J., Comerford A.P., McLaren C.A., Butler C.R., Crowley C., McIntyre D., Sebire N.J., Janes S.M., O’Callaghan C., Mason C., De Coppi P., Lowdell M.W., Elliott M.J., Birchall M.A. (2015). Tissue-engineered tracheal replacement in a child: a 4-year follow-up study. Am. J. Transpl..

[8] Macchiarini P., Jungebluth P., Go T., Asnaghi M.A., Rees L.E., Cogan T.A., Dodson A., Martorell J., Bellini S., Parnigotto P.P., Dickinson S.C., Hollander A.P., Mantero S., Conconi M.T., Birchall M.A. (2008). Clinical transplantation of a tissue-engineered airway. Lancet.

[9] Jungebluth P., Alici E., Baiguera S., Le Blanc K., Blomberg P., Bozoky B., Crowley C., Einarsson O., Grinnemo K.H., Gudbjartsson T., Le Guyader S., Henriksson G., Hermanson O., Juto J.E., Leidner B., Lilja T., Liska J., Luedde T., Lundin V., Moll G., Nilsson B., Roderburg C., Stromblad S., Sutlu T., Teixeira A.I., Watz E., Seifalian A., Macchiarini P. (2011). Tracheobronchial transplantation with a stem-cell-seeded bioartificial nanocomposite: a proof-of-concept study. Lancet.

[10] Berg M., Ejnell H., Kovacs A., Nayakawde N., Patil P.B., Joshi M., Aziz L., Radberg G., Hajizadeh S., Olausson M., Sumitran-Holgersson S. (2014). Replacement of a tracheal stenosis with a tissue-engineered human trachea using autologous stem cells: a case report. Tissue Eng. Pt A.

[11] Butler C.R., Hynds R.E., Gowers K.H., Lee D.D.H., Brown J.M., Crowley C., Teixeira V.H., Smith C.M., Urbani L., Hamilton N.J., Thakrar R.M., Booth H.L., Birchall M.A., De Coppi P., Giangreco A., O’Callaghan C., Janes S.M. (2016). Rapid expansion of human epithelial stem cells suitable for airway tissue engineering. Am. J. Respir. Crit. Care Med..

[12] Huang S.X., Islam M.N., O’Neill J., Hu Z., Yang Y.G., Chen Y.W., Mumau M., Green M.D., Vunjak-Novakovic G., Bhattacharya J., Snoeck H.W. (2014). Efficient generation of lung and airway epithelial cells from human pluripotent stem cells. Nat. Biotechnol..

[13] Mou H., Zhao R., Sherwood R., Ahfeldt T., Lapey A., Wain J., Sicilian L., Izvolsky K., Musunuru K., Cowan C., Rajagopal J. (2012). Generation of multipotent lung and airway progenitors from mouse ESCs and patient-specific cystic fibrosis iPSCs. Cell Stem Cell.

[14] Wong A.P., Chin S., Xia S., Garner J., Bear C.E., Rossant J. (2015). Efficient generation of functional CFTR-expressing airway epithelial cells from human pluripotent stem cells. Nat. Protoc..

[15] Wong A.P., Bear C.E., Chin S., Pasceri P., Thompson T.O., Huan L.J., Ratjen F., Ellis J., Rossant J. (2012). Directed differentiation of human pluripotent stem cells into mature airway epithelia expressing functional CFTR protein. Nat. Biotechnol..

[16] Firth A.L., Dargitz C.T., Qualls S.J., Menon T., Wright R., Singer O., Gage F.H., Khanna A., Verma I.M. (2014). Generation of multiciliated cells in functional airway epithelia from human induced pluripotent stem cells. Proc. Natl. Acad. Sci. U. S. A..

[17] Jungebluth P., Alici E., Baiguera S., Blomberg P., Bozoky B., Crowley C., Einarsson O., Gudbjartsson T., Le Guyader S., Henriksson G., Hermanson O., Juto J.E., Leidner B., Lilja T., Liska J., Luedde T., Lundin V., Moll G., Roderburg C., Stromblad S., Sutlu T., Watz E., Seifalian A., Macchiarini P. (2011). Tracheobronchial transplantation with a stem-cell-seeded bioartificial nanocomposite: a proof-of-concept study. Lancet.

[18] Zhao L., Sundaram S., Le A., Huang A., Zhang J., Hatachi G., Berkolaisev A., Caty M.G., Yi T., Leiby K.L., Gard A.L., Kural M.H., Gui L., Rocco K.A., Sivarapatna A., Calle E.A., Greaney A., Urbani L., Maghsoudlou P., DeCoppi P., Burns A., Niklason L. (2016). Engineered tissue-stent biocomposites as tracheal replacements. Tissue Eng. Part A.

[19] Hoshiba T., Lu H., Kawazoe N., Chen G. (2010). Decellularized matrices for tissue engineering. Expert Opin. Biol. Ther..

[20] Tapias L.F., Ott H.C. (2014). Decellularized scaffolds as a platform for bioengineered organs. Curr. Opin. Organ Transpl..

[21] Crapo P.M., Gilbert T.W., Badylak S.F. (2011). An overview of tissue and whole organ decellularization processes. Biomaterials.

[22] Basu J., Ludlow J.W. (2010). Platform technologies for tubular organ regeneration. Trends Biotechnol..

[23] Conconi M.T., De Coppi P., Di Liddo R., Vigolo S., Zanon G.F., Parnigotto P.P., Nussdorfer G.G. (2005). Tracheal matrices, obtained by a detergent-enzymatic method, support in vitro the adhesion of chondrocytes and tracheal epithelial cells. Transpl. Int. Off. J. Eur. Soc. Organ Transplant..

[24] van Veenendaal M.B., Liem K.D., Marres H.A. (2000). Congenital absence of the trachea. Eur. J. Pediatr..

[25] Maughan E., Lesage F., Butler C.R., Hynds R.E., Hewitt R., Janes S.M., Deprest J.A., Coppi P.D. (2016). Airway tissue engineering for congenital laryngotracheal disease. Semin. Pediatr. Surg..

[26] Lange P., Greco K., Partington L., Carvalho C., Oliani S., Birchall M.A., Sibbons P.D., Lowdell M.W., Ansari T. (2015). Pilot study of a novel vacuum-assisted method for decellularization of tracheae for clinical tissue engineering applications. J. Tissue Eng. Regen. Med..

[27] Aamodt J.M., Grainger D.W. (2016). Extracellular matrix-based biomaterial scaffolds and the host response. Biomaterials.

[28] Baiguera S., Jungebluth P., Burns A., Mavilia C., Haag J., De Coppi P., Macchiarini P. (2010). Tissue engineered human tracheas for in vivo implantation. Biomaterials.

[29] Maghsoudlou P., Georgiades F., Tyraskis A., Totonelli G., Loukogeorgakis S.P., Orlando G., Shangaris P., Lange P., Delalande J.M., Burns A.J., Cenedese A., Sebire N.J., Turmaine M., Guest B.N., Alcorn J.F., Atala A., Birchall M.A., Elliott M.J., Eaton S., Pierro A., Gilbert T.W., De Coppi P. (2013). Preservation of micro-architecture and angiogenic potential in a pulmonary acellular matrix obtained using intermittent intra-tracheal flow of detergent enzymatic treatment. Biomaterials.

[30] Fulcher M.L., Gabriel S., Burns K.A., Yankaskas J.R., Randell S.H. (2005). Well-differentiated human airway epithelial cell cultures. Methods Mol. Med..

[31] Crowley C., Klanrit P., Butler C.R., Varanou A., Plate M., Hynds R.E., Chambers R.C., Seifalian A.M., Birchall M.A., Janes S.M. (2016). Surface modification of a POSS-nanocomposite material to enhance cellular integration of a synthetic bioscaffold. Biomaterials.

[32] Loebinger M.R., Eddaoudi A., Davies D., Janes S.M. (2009). Mesenchymal stem cell delivery of TRAIL can eliminate metastatic cancer. Cancer Res..

[33] Totonelli G., Maghsoudlou P., Garriboli M., Riegler J., Orlando G., Burns A.J., Sebire N.J., Smith V.V., Fishman J.M., Ghionzoli M., Turmaine M., Birchall M.A., Atala A., Soker S., Lythgoe M.F., Seifalian A., Pierro A., Eaton S., De Coppi P. (2012). A rat decellularized small bowel scaffold that preserves villus-crypt architecture for intestinal regeneration. Biomaterials.

[34] Hamilton N., Bullock A.J., Macneil S., Janes S.M., Birchall M. (2014). Tissue engineering airway mucosa: a systematic review. Laryngoscope.

[35] Zhang H., Fu W., Xu Z. (2015). Re-epithelialization: a key element in tracheal tissue engineering. Regen. Med..

[36] Ribatti D., Nico B., Vacca A., Presta M. (2006). The gelatin sponge-chorioallantoic membrane assay. Nat. Protoc..

[37] Londono R., Badylak S.F. (2015). Biologic scaffolds for regenerative medicine: mechanisms of in vivo remodeling. Ann. Biomed. Eng..

[38] Baiguera S., Del Gaudio C., Jaus M.O., Polizzi L., Gonfiotti A., Comin C.E., Bianco A., Ribatti D., Taylor D.A., Macchiarini P. (2012). Long-term changes to in vitro preserved bioengineered human trachea and their implications for decellularized tissues. Biomaterials.

[39] Partington L., Mordan N.J., Mason C., Knowles J.C., Kim H.W., Lowdell M.W., Birchall M.A., Wall I.B. (2013). Biochemical changes caused by decellularization may compromise mechanical integrity of tracheal scaffolds. Acta Biomater..

[40] Faulk D.M., Carruthers C.A., Warner H.J., Kramer C.R., Reing J.E., Zhang L., D’Amore A., Badylak S.F. (2014). The effect of detergents on the basement membrane complex of a biologic scaffold material. Acta Biomater..

[41] Pellegrini G., Ranno R., Stracuzzi G., Bondanza S., Guerra L., Zambruno G., Micali G., De Luca M. (1999). The control of epidermal stem cells (holoclones) in the treatment of massive full-thickness burns with autologous keratinocytes cultured on fibrin. Transplantation.

[42] Ronfard V., Rives J.M., Neveux Y., Carsin H., Barrandon Y. (2000). Long-term regeneration of human epidermis on third degree burns transplanted with autologous cultured epithelium grown on a fibrin matrix. Transplantation.

[43] Pellegrini G., Traverso C.E., Franzi A.T., Zingirian M., Cancedda R., De Luca M. (1997). Long-term restoration of damaged corneal surfaces with autologous cultivated corneal epithelium. Lancet.

[44] Hynds R.E., Giangreco A. (2013). Concise review: the relevance of human stem cell-derived organoid models for epithelial translational medicine. Stem Cells.

[45] Kutten J.C., McGovern D., Hobson C.M., Luffy S.A., Nieponice A., Tobita K., Francis R.J., Reynolds S.D., Isenberg J.S., Gilbert T.W. (2015). Decellularized tracheal extracellular matrix supports epithelial migration, differentiation, and function. Tissue Eng. Part A.

[46] Pageau S.C., Sazonova O.V., Wong J.Y., Soto A.M., Sonnenschein C. (2011). The effect of stromal components on the modulation of the phenotype of human bronchial epithelial cells in 3D culture. Biomaterials.

[47] Weiss D.J., Elliott M., Jang Q., Poole B., Birchall M. (2014). C. International society of cell therapy pulmonary scientific, tracheal bioengineering: the next steps. Proceeds of an International Society of Cell Therapy Pulmonary Cellular Therapy Signature Series Workshop, Paris, France, April 22, 2014, Cytotherapy 16(12).

